# Management of bleeding from morbidly adherent placenta during elective repeat caesarean section: retrospective -record -based study

**DOI:** 10.1186/s12884-019-2244-4

**Published:** 2019-03-29

**Authors:** Saad El Gelany, Emad M. Ibrahim, Mo’men Mohammed, Ahmed R. Abdelraheim, Eissa M. Khalifa, Ahmed K. Abdelhakium, Ayman M. Yousef, Heba Hassan, Khaled Goma, Mohammed Khairy

**Affiliations:** Obstetrics and Gynecology Department, Faculty of Medicine, Minia Maternity and Children University Hospital, Minia University, Elsalam, Eloboor, Maghaghaga City, Minya Egypt

**Keywords:** Morbidly adherent placenta, Cervix, Natural tamponade, Major obstetric haemorrhage

## Abstract

**Background:**

Controlling massive haemorrhage from morbidly adherent placenta (MAP) at caesarean section is a major surgical challenge to obstetricians. This study compares different intra-operative interventions to control haemorrhage from morbidly adherent placenta and its impact on maternal morbidity.

**Methods:**

Retrospective analysis was done for baseline characteristics, intra-operative and postoperative complications of 125 patients with morbidly adherent placenta who had elective CS at 35–38 weeks gestation in the period from 01/2012 to 01/2017. The included patients were categorized into three groups according to intra-operative interventions they had for controlling bleeding; Group A (*n* = 42) had only balloon tamponade, Group B (*n* = 40) had balloon tamponade and bilateral uterine artery ligation, in Group C (*n* = 43) all cases were managed by bilateral uterine artery ligation and inverting the cervix into the uterine cavity and suturing the anterior and/or the posterior cervical lips into the anterior and/or posterior walls of the lower uterine segment using the cervix as a natural tamponade.

**Results:**

There were no differences of baseline characteristics of patients in all groups. Group C had significantly better outcomes as compared with groups A and B; less total blood loss (Group C 2869.5 ml vs Group B 4580 ml, Group A 4812 ml, *P* <  0.001), less requirement of blood transfusion more than 4 units (Group C 4/43, Group B 10/40,Group A 12/42, *P* <  0.02), significant reduction in prolonged hospital stay over 10 days (Group C 2/43, Group B 9/40,Group A 14/42, *P* < 0.001) and lower risk of coagulopathy (Group C 4/43, B 8/40, A 9/42), visceral injuries (Group C 4/43 vs B 8/40, A 10/42,*P* < 0.01) and need for hysterectomy (Group C 4/43 vs B 11/40, A 13/42,P < 0.001).

**Conclusion:**

A combination bilateral uterine artery ligation and using the cervix as a natural tamponade are very effective and simple methods in controlling bleeding resulting from separated placenta accreta.

**Trial registration:**

The findings are part of the research project registered in ClinicalTrials.gov NCT02590484. Registered 28 October 2015**.**

**Electronic supplementary material:**

The online version of this article (10.1186/s12884-019-2244-4) contains supplementary material, which is available to authorized users.

## Background

Morbidly adherent placenta (MAP) is one of the major causes of massive obstetric haemorrhage. It is a rare but potentially life-threatening complication of pregnancy. The steady rise of caesarean section (CS) delivery rates in recent years is associated with increasing incidence of both placenta previa and placenta accreta [1].The incidence of placenta accreta in the presence of placenta Previa increases from 24% after one caesarean section to 67% after four or more caesarean sections [2].

The optimal management of placenta accreta spectrum remains controversial. Whilst hysterectomy or conservative management are recommended in case of confirmed MAP during caesarean section [3] Intraoperative bleeding may occur due to either partial separation of the placenta following a tentative attempt to confirm diagnosis clinically or in cases of unconfirmed partial accreta.

There is lack of consensus on the optimal uterine sparing surgical approach to reduce intraoperative bleeding if MAP is partially separated. Whilst electing for timely hysterectomy may be recommended and lifesaving [3] this may not be ideal for women wishing to preserve their fertility and uterine sparing alternative interventions are highly needed.

As intraoperative bleeding from MAP is often massive and dramatically quick resulting in severe maternal morbidity and mortality it is of utmost importance to have a pre-planned approach to this surgical challenge that is effective and swift.

In this study we report on our own centre experience of three procedures to try and reduce intraoperative bleeding and report on its effects on maternal morbidity and mortality postoperatively.

## Methods

This is a retrospective study reporting on patients with suspected MAP who had a repeat elective CS in Minia maternity university Hospital, Egypt in the period from 01/01/2012 to 01/01/2017.

The interventions in the study as a part of the trial that was registered on ClinicalTrials.gov NCT02590484. Registered 28 October 2015.

### Inclusion criteria

**Fig. 1:**
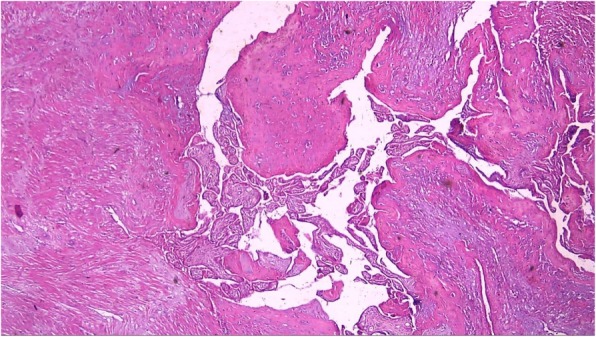
showed site of closely attached chorionic villi to the myometrium; stained by hematoxylin and eosin × 100

Patients included in this study were women with at least one previous caesarean section and placenta previa with suspect MAP who were keen to preserve their fertility if possible and were booked for elective repeat caesarean section. Cases were only included if partial separation occurred at CS resulting in major bleeding. The diagnosis of MAP was confirmed by histopathological study of the removed part of the placenta showing deep invasion of chorionic villi and presence of myometrial fibres (Fig. 1).

### Exclusion criteria

Patients who had previous CS/ placenta praevia only with no features of MAP were excluded from this study. We also excluded patients with previous CS with placenta praevia/ accreta who required an emergency caesarean section due to major antepartum haemorrhage (APH) and patients who had preoperative diagnosis of placenta percreta who opted to have an elective hysterectomy or when placenta percreta is confirmed intraoperatively.

The primary outcome for the study was the total volume of blood loss in the intra and postoperative period and the need for hysterectomy. The secondary outcomes were maternal morbidities composite including coagulopathy, need for massive blood transfusion (> 4 units),length of hospital stay > 10 days, and visceral injuries.Other secondary outcomes intended to be reported were maternal mortalities if any present and gynaecological complications as amenorrhoea,intrauterine adhesions.

### Diagnostic criteria

The ultrasound features used for suspicion of MAP were as described previously in literature including one or more of the following;

Loss or thinning (‹1 mm) of the normal hypo-echoic retro-placental myometrial plane or thinning or disruption of the hyper-echoic uterine serosa bladder interface or presence of multiple placental lakes [4].

Patients with equivocal ultrasound diagnosis placenta accreta or suspicion of placenta percreta had magnetic resonant imaging (MRI) scan to confirm or refute the diagnosis.

The MRI features used to diagnose placenta accreta/percreta were as described previously; uterine bulging, heterogeneous signal intensity within placenta, dark intra-placental bands, focal interruption to myometrial wall,invasion of pelvic structures by placental tissue [5].

### Management protocol

All patients fulfilling the inclusion criteria had the following protocol of antenatal and intrapartum management.

#### Antenatal management

All patients with suspected MAP included in this study were admitted to inpatient department for close observations if they had a minor APH and administration of corticosteroids if < 34 weeks gestation. Patients were given hematinic medications during their admission to keep their haemoglobin level above 12 g/dl. At least 4 Cross matched blood units were always kept available. They were booked for elective repeat CS at 37-38 weeks if they remain asymptomatic or their elective CS brought forward to 35–36 weeks if they had any further minor APH.

All patients were fully counselled regarding the risk of bleeding and surgical options at delivery if placenta accreta is confirmed including risk of hysterectomy.

#### Operative protocol

During the CS, the patients were either in supine or lithotomy position, then opening the skin with a vertical midline or transverse incision. This is followed by opening the anterior abdominal wall in layers. The urinary bladder was dissected downward. For most cases lower segment CS incision was used unless unexpected percreta was noted intraoperatively. After delivery of the foetus a short tentative attempt of delivery of the placenta was performed if it was a suspect MAP to confirm diagnosis. This was not done however if placenta percreta was confirmed intra-operatively.

In the majority of patients encountered in our series we have not encountered a total placenta accreta where partial separation of the placenta is not possible.

In case of partially separated placenta accreta with ensuing bleeding, further action depended on patient’s fertility wishes and elective hysterectomy was done for patients who completed their family however; for patients who wished to preserve their fertility prompt blood transfusion, available uterotonic agents were injected. Patients had one of three intraoperative surgical interventions that evolved during the study period with group A (balloon Tamponade only) being the earliest group and group C being the most recent cases. There was however some chronological overlap over these groups depending on the surgeon’s preference and expertise.

These procedures were used as primary surgical strategy to control bleeding however in case of continued bleeding from the placental bed; a timely emergency hysterectomy was done. Therefore, patients in this study were divided into three groups according to the intraoperative intervention to control bleeding;

##### Group A

In which patients had only Bakri Balloon inserted transabdominally through the CS incision to tamponade the placenta bed in the lower uterine segment after inflation of the balloon with 250 ml of saline and the balloon tubes were brought vaginally with vaginal packs inserted to keep the balloon in situ and avoid early expulsion. Uterus was closed over the balloon which was kept in for 24 h and removed postoperatively in operating theatre. [6]

##### Group B

In which patients had bilateral uterine artery ligations as described before [7, 8]. Briefly two large vicryl stitches were passed using a large size 3/8 needle below and lateral to the lower edge of the uterine incision angle in anteroposterior direction and then redirected from back to the front through avascular window in the posterior leaf of the broad ligament just lateral to the uterine border taking care to avoid injury to bowel posteriorly or bladder/ureter anteriorly. The stitches were tied securely anteriorly. This was followed by insertion of the Balloon tamponade as in group A.

##### Group C

In this group, a combination of bilateral uterine artery ligations and cervical tamponade by elevating the cervix into the uterine cavity using Allis forceps then suturing the anterior and/or posterior cervical lip (s) into the anterior and /or posterior uterine segment (s) depending upon the site of bleeding by two or three simple interrupted stitches. Hegar dilator was inserted from the uterine cavity to confirm patency of the cervical canal. Closure of the uterine incision after control of bleeding (Additional file 1).


**Additional file 1: Movie S1** Cervical tamponade in placenta accreta. This is a movie file showing detailed steps of cervical tamponade in which the cervical lips are used to control bleeding in placenta accreta. The video was recorded by the authors of this research article. (MP4 12121 kb)


In group C, Follow up appointments at 3 and 6 months was given after delivery where revision of history, clinical, speculum and ultrasound examinations was performed.

In all patients preoperative, intraoperative and postoperative data were collected and used in this report.

### Statistical analysis

The collected data were statistically analysed using SPSS software version 20 (Statistical Package for Social Sciences, IBM, USA).

Descriptive statistics were done for continuous variables using mean, standard deviation, while they were done for categorical data by number and percentage.

Comparisons for continuous variables were done using one-way ANOVA test and post Hoc Tukey’s Correction and Chi square test was used for categorical variables between groups when the cell contains more than 5, and Fisher exact test when the cell contains less than 5. The level of significance was taken at (*P* value ≤0.05).

## Results

In this study 125 patients fulfilled the inclusion criteria during our study period. Group A (balloon Tamponade only) included 42 patients, Group B (balloon Tamponade and uterine artery ligation) 40 patients and group C (uterine artery ligation and cervical tamponade) included 43 patients.

There were no statistically significant differences in the baseline characteristics (age, BMI, parity, number of previous caesarean sections, type of placenta previa, location of placenta previa, gestational age at delivery and preoperative haemoglobin) of the patients in the three intervention groups A, B and C as shown in Table 1.

**Table 1:** Baseline characteristics of patients with placenta previa/ accreta in the tree groups

	Group A (*n* = 42)	Group B (*n* = 40)	Group C (*n* = 43)	*P* value
Age (yrs)	25.15 ± 5.13	26.75 ± 6.01	25.75 ± 6.39	NS
Height (cm)	162.5 ± 6.66	164.05 ± 7.99	165.3 ± 7.92	NS
Weight (kg)	70.45 ± 6.96	69 ± 6.57	70.15 ± 6.18	NS
BMI (Kg/m2)	27.3 ± 4.2	26.8 ± 3.5	26.5 ± 2.5	NS
Parity	2.4 ± 1.3	2.5 ± 1.6	2.7 ± 1.2	NS
No. of CS				
One	11 (55%)	12 (60%)	11 (55%)	
Two	8 (40%)	7 (35%)	8 (40%)	
Three or more	1 (5%)	1 (5%)	1(10%)	
Gestational age at delivery (weeks)	37.55 ± 2.18	38.1 ± 2.05	37.8 ± 1.82	NS
Preoperative haemoglobin (g/dl)	11.75 ± 0.52	11.52 ± 0.44	11.59 ± 0.55	NS
Placenta previa				
Minor	11 (26.2%)	6 (15%)	8 (18.6%)	NS
Major	31 (73.8%)	43 (85%)	35 (81.4%)	
Placental site				
Anterior	36 (85.7%)	36 (90%)	38 (88.4%)	NS
posterior	6 (14.3%)	4 (10%)	5 (11.6%)	

*NS* Not significant

Minor placenta previa includes grade I and grade II placenta previa. Grade I placenta previa is defined as a lower edge inside the lower uterine segment; grade II or marginal previa as a lower edge reaching the internal os

Major placenta previa includes grade III and grade IV placenta previa. Grade III or partial previa when the placenta partially covers the cervix; and grade IV or complete previa when the placenta completely covers the cervix

Table 2 shows intra operative and early postoperative observations for the three intervention groups. Group C has the least surgical operation time. Also, the total estimated blood loss (intraoperative and postoperative) was significantly less in Group C compared with Groups A and B (*P* < 0.001).

**Table 2:** Intra operative and early postoperative observations for the three intervention groups

Duration of surgery (minutes)	Group A	Group B	Group C	*P* value
	86.85 ± 4.68	84.7 ± 2.36	84.65 ± 3.26^a^	NS
Intraoperative vital signs^╪^				
HR	105.25 ± 6.36	102.25 ± 6.51	102.6 ± 6.61	NS
RR	18.9 ± 1.86	18.7 ± 1.86	18.5 ± 1.48	NS
Systolic BP	123.8 ± 10.43	118.4 ± 11.21	121.65 ± 9.89	NS
Diastolic BP	69.75 ± 6.47	70.2 ± 6.82	68.6 ± 5.79	NS
Intraoperative Blood loss (ml) Placenta-CS	3685.5 ± 108.55	3427 ± 30.3	2856.5 ± 21.73^b^	< 0.001^a^
PostCS-2 h blood loss (ml)	632.65 ± 9.6	610.35 ± 34.43	101.3 ± 34.23^c^	< 0.001^b^
Total Blood loss (ml)	4812 ± 111.3	4580 ± 48.2	2869.5 ± 38.38^d^	< 0.001^c^

^╪^ Values presented as mean and SEM

^a^One way ANOVA (F = 10.6 *P* < 0.001, Group C vs A F = 10.3 post Hoc Tukey *P* < 0.001,Group C vs Group B F = 10.2post Hoc Tukey *P* < 0.01 Group A vs Group B F = 1.72 *P* = 0.06)

^b^One way ANOVA (F = 11.2 *P* < 0.001, Group C vs A F = 11.5 post Hoc Tukey *P* < 0.001,Group C vs Group B F = 11.1 post Hoc Tukey P < 0.001 Group A vs Group B F = 0.67 *P* = 0.2)

^c^One way ANOVA (F = 10.2 *P* < 0.001, Group C vs A F = 10.8 post Hoc Tukey *P* < 0.001,Group C vs Group B F = 10.1 post Hoc Tukey *P* < 0.001 Group A vs Group B F = 0.93 *P* = 0.07)

Table 3 shows postoperative morbidities in patients with placenta accreta undergoing different procedures. Group C has significantly less need for major blood transfusion and significantly less incidence of coagulopathy. The number of patients requiring admission to ICU and hospitalisation for > 10 days was significantly less in Group C compared to groups A and B. Bladder injuries in Group C were significantly less in Group C compared with Groups A and B. Also, the need for hysterectomy was significantly less in Group C compared with groups A and B. Therefore, the overall morbidity was less and the recovery was quicker for cases in group C was less than the other two groups. There were no maternal mortalities in all groups.

**Table 3:** Postoperative morbidities in patients with placenta accreta undergoing different procedures

	Group A *N* = 42	Group B *N* = 40	Group C *N* = 43	*P* value
Coagulopathy	9 (21.4%)	8 (20%)	4 (9.3%)	0.03^b^
Blood transfusion > 4 packed RBC	12 (28.6%)	10 (25%)	4(9.3%)	0.02^c^
ICU admission	5 (22.7%)	8 (20%)	3 (7%)	0.03^d^
Hospital stay > 10 days	14 (33.3%)	9 (22.5%)	2(4.6%)	0.001^e^
Bladder injuries	10 (23.8%)	8 (20%)	4 (9.3%)	0.01^f^
Hysterectomy	13 (30.9%)	11 (27.5%)	4(9.3%)	0.001^g^

All values presented indicate number of patients affected by each morbidity and its percentage in the group

^b^Chi Square test overall 0.03

^c^Chi square test overall 0.02 Group C vs Group A *P* < 0.001,Group C vs Group B *P* = 0.001, Group A vs Group B P 0.04

^d^Fisher Exact test overall 0.03 Group C vs Group A *P* < 0.001,Group C vs Group B *P* = 0.001, Group A vs Group B *P* = 0.1

^e^Chi square test overall *P* = 0.001 Group C vs Group A *P* < 0.001,Group C vs Group B *P* = 0.007, Group A vs Group B *P* = 0.05

^f^_Chi square test overall *P* = 0.01 Group C vs group A P < 0.001,Group C vs group B *P* < 0.01, group A vs group B *P* = 0.07_

^g^Chi square test overall *p* = 0.001 Group C vs Group A *P* < 0.001,Group C vs Group B *P* = 0.005, Group A vs Group B *P* = 0.04

In group C, at 3 months appointment, there were no remarkable clinical, speculum and ultrasound findings in 37 cases. In two patients, the cervical lips were displaced upwards with no pathological coloposcopic and/or hysterocopic findings. Four cases lost follow-up.

Thirty five patients (81.4%) were seen at 6 months appointment, where menstruation was resumed in 33 (76.7%) patients while the other ten patients (23.3%) were amenorrheic that could be explained by lactation or another cause and currently they are under follow-up. Contact details of the hospital and research coordinator(s) were given to the patients in case of experiencing unusual symptoms.

## Discussion

This study has shown that prompt use of a combination of bilateral uterine artery ligation and cervical tamponade are simple, cost effective and most effective ways of controlling bleeding due to MAP and has led to significant reduction of maternal morbidity and need for hysterectomy.

Women included in this report were very keen to preserve their fertility and the options that were acceptable to them were either uterine sparing surgical interventions or conservative management. The latter approach (cutting the cord short and leaving placenta in situ) though has recently been recommended [3, 9], was not to feasible in our series due to ensuing bleeding following partial separation of the placenta. Furthermore, the limitation of our experience with conservative management of placenta accreta with uncertainty about risks of sepsis, secondary haemorrhage and later need for hysterectomy as well as the need for lengthy follow-up by compliant patients and possible long-term effect on fertility and gestational trophoblastic disease had made this option less favourable in our own setting.

A number of other studies have reported on the use of various interventions to stop bleeding following partial separation of MAP. These included preoperative insertion of uterine artery catheters to embolise blood vessels in the placental bed [10], internal iliac artery ligation [11], suturing the cervix to the placental bed to occlude bleeding surface [12, 13], the B-Lynch suture [14], insertion of parallel vertical compression sutures [15], a square suturing technique [16], use of multiple 8 compression suturing as a novel procedure to preserve fertility in patients with placenta accreta [17] and Triple-P procedure [perioperative placental localisation, pelvic devascularisation and placental non-separation] involving delivery of the fetus via transverse uterine incision above the upper border of the placenta, myometrial excision and reconstruction of the uterine wall [18, 19] It is likely that there is no single intervention alone will be enough to control the massive bleeding ensuing after separation of MAP. As demonstrated in this study using a combination of the techniques of Balloon tamponade, uterine artery ligation and cervical inversion techniques has been shown to be most effective way of controlling bleeding after MAP.

In 2007, Dawlatly and his colleagues published a case report in which they used the inverted cervical lip(s) to control bleeding from the placental bed and they succeeded to preserve the uterus and patient life [12]. Another study supporting our findings done by Sakhavar et al. who exerts a temporary pressure on the lower segment arteries by using cervical inversion thus reducing blood flow leading to relative hemostasis, after which the placental bed is sutured to control bleeding. After bleeding is controlled, the cervix is returned again to its original position. In such case series, the bleeding was stopped within 3–5 min with no reported major complications, blood transfusions or obstetric hysterectomies [20].

We believe that these techniques should be used as a primary line surgical intervention for controlling bleeding due to MAP. The techniques described in this report are readily available in most settings, easy to learn and apply and do not require sophisticated equipment as in uterine artery embolization or special skills and expertise as in ligation of internal iliac artery. These surgical measures should be provided as a part of a comprehensive preoperative and intraoperative care bundle. We suggest using a stepwise approach of intraoperative control of bleeding utilizing combination of uterine artery ligation, balloon tamponade and use cervical inversion as first step. This can be escalated to other techniques as internal iliac artery ligation and/or uterine artery embolization if first line measures are inadequate with prompt timely recourse to hysterectomy as a life-saving measure if all other measures proved futile.

The strenghths of our study is its comprehensive preoperative diagnostic work-up and comprehensive reporting on techniques with significant impact on maternal outcomes and relatively large sample size for this uncommon obstetric problem. Our study is limited by its retrospective nature which could have introduced an element of performance bias. This may have led to the apparent shortening of operating time in the group C as these patients were the most recent group with possible better performance as experience accumulating in dealing with similar emergencies in the other two groups. Although all groups were not significantly different in known baseline confounding variables it is possible that with accumulating radiological experience and more confident diagnosis of MAP, Ultrasound -confirmed cases had more directed counselling for hysterectomy leaving only suspected cases with partial accreta in the late stages of study period. This may have introduced an element of selection bias.

A prospective controlled study to address these limitations however due to the uncommon nature of the problem and impact of other factors (as patient’s preference and clinician’s/centre experience and local protocols) would be difficult to conduct.

## Conclusions

The combination of bilateral uterine artery ligation and using the cervix as a natural tamponade are very effective and simple methods in controlling bleeding resulting from partially separated MAP. Use of these surgical techniques in combination with other preoperative and intraoperative measures have led to less risk of maternal morbidities.

## Abbreviations

APS: Antepartum haemorrhage
CS: Caesarean section
MAP: Morbidly adherent placenta
MRI: Magnetic resonant imaging
PPH: Postpartum haemorrhage
